# The concept of physical limitations in knee osteoarthritis – the view of patients and health professionals

**DOI:** 10.1186/1745-6215-16-S1-P29

**Published:** 2015-05-29

**Authors:** Louise Klokker, Richard H Osborne, Eva Ejlersen Waehrens, Ole Norgaard, Elisabeth Bandak, Henning Bliddal, Marius Henriksen

**Affiliations:** 1 The Parker Institute, Copenhagen University Hospital, Bispebjerg & Frederiksberg, Copenhagen, Denmark; 2 Population Health Strategic Research Centre, School of Health and Social Development, Faculty of Health, Deakin University, Burwood, Australia; 3 Department of Public Health, Faculty of Health and Medical Sciences, University of Copenhagen, Denmark; 4 The Research Initiative for Activity studies and Occupational Therapy, Institute of Public Health, University of Southern Denmark, Odense, Denmark

## Background

Recommendations of core outcomes in clinical trials on knee osteoarthritis (OA) include ‘physical function’ but no definition is provided. The objective of this study was to comprehensively identify components of the ‘physical limitation’ concept in knee OA, and to rate the clinical importance of these by using the perspective of both patients and health professionals.

## Method

Concept Mapping, a structured group process, was used to identify and organize components of the ‘physical limitation’ concept. Statements were generated through workshops with patients and through e-mail and an international web-based survey with health professionals. Ideas were elicited through a nominal group technique and organized using multidimensional scaling, hierarchical cluster analysis, participant validation, rating of clinical importance, and thematic analyses, to generate a conceptual model of physical limitations in knee OA.

## Results

Fifteen Danish patients and 200 international professionals contributed, producing 1739 statements. Omitting redundancies, 361 individual statements were thematically grouped by participants. Five clusters emerged: ‘Limitations/physical deficits’; ‘Everyday hurdles’; ‘You’re not the person you used to be’; ‘Need to adjust way of living’ and ‘External limitations’, each with sub-clusters. Twelve sub-clusters were rated significantly more important by patients, and one was rated higher by professionals.

## Conclusion

Patients and professionals agreed largely on the physical limitation concept in knee OA. Some limitations of high importance to patients were underestimated by the professionals, highlighting the importance of patient involvement. These data offer new knowledge to guide selection of clinically relevant outcomes and development of outcome measures in knee OA.

